# Stabilization of low-cost phase change materials for thermal energy storage applications

**DOI:** 10.1016/j.isci.2023.107175

**Published:** 2023-06-20

**Authors:** Damilola O. Akamo, Navin Kumar, Yuzhan Li, Collin Pekol, Kai Li, Monojoy Goswami, Jason Hirschey, Tim J. LaClair, David J. Keffer, Orlando Rios, Kyle R. Gluesenkamp

**Affiliations:** 1 The Bredesen Center for Interdisciplinary Research and Graduate Education, University of Tennessee, Knoxville, TN 37996, USA; 2 Building Energy Efficiency Group, Gas Technology Institute, Des Plaines, IL 60018, USA; 3 School of Materials Science and Engineering, University of Science and Technology Beijing, Beijing 100081, China; 4 Materials Science and Engineering Department, University of Tennessee, Knoxville, TN 37996, USA; 5 Buildings and Transportation Sciences Division, Oak Ridge National Laboratory, Oak Ridge, TN 37830, USA; 6 George W. Woodruff School of Mechanical Engineering, Georgia Institute of Technology, Atlanta, GA, USA; 7 Building Energy Science Group, National Renewable Energy Laboratory, Golden, CO 80401, USA; 8 Chemical Sciences Division, Oak Ridge National Laboratory, Oak Ridge, TN 37830, USA

**Keywords:** Phase transformation, Materials science, Materials application, Energy materials

## Abstract

Sodium sulfate decahydrate (Na_2_SO_4_^.^10H_2_O, SSD), a low-cost phase change material (PCM), can store thermal energy. However, phase separation and unstable energy storage capacity (ESC) limit its use. To address these concerns, eight polymer additives—sodium polyacrylate (SPA), carboxymethyl cellulose (CMC), Fumed silica (SiO_2_), potassium polyacrylate (PPA), cellulose nanofiber (CNF), hydroxyethyl cellulose (HEC), dextran sulfate sodium (DSS), and poly(sodium 4-styrenesulfonate) (PSS)—were used to explore several stabilization mechanisms. The ESC of PCMs deteriorated when thickeners, SPA, PPA, and CNF, were added. DSS-modified PCMs exhibited greater stability up to 150 cycles. Rheology measurements indicated that DSS did not impact SSD viscosity significantly during stabilization. Dynamic light scattering showed that DSS reduces SSD particle size and electrostatically suspends salt particles in a stable homogeneous solution, avoiding phase separation. This study proposes a promising method to improve the thermal stability of salt hydrate PCMs by utilizing polyelectrolyte-salt hydrate mixture for thermal energy storage applications.

## Highlights

• Stabilization of sodium sulfate decahydrate (SSD) using polymeric additives
• Evaluation of structural, thermal, and rheological properties of PCM composites
• Improved thermal cycling stability up to 150 cycles using polyelectrolyte
• Polyelectrolyte-stabilized salt hydrate for long-term thermal stability of PCMs

## Graphical abstract

**Figure:**
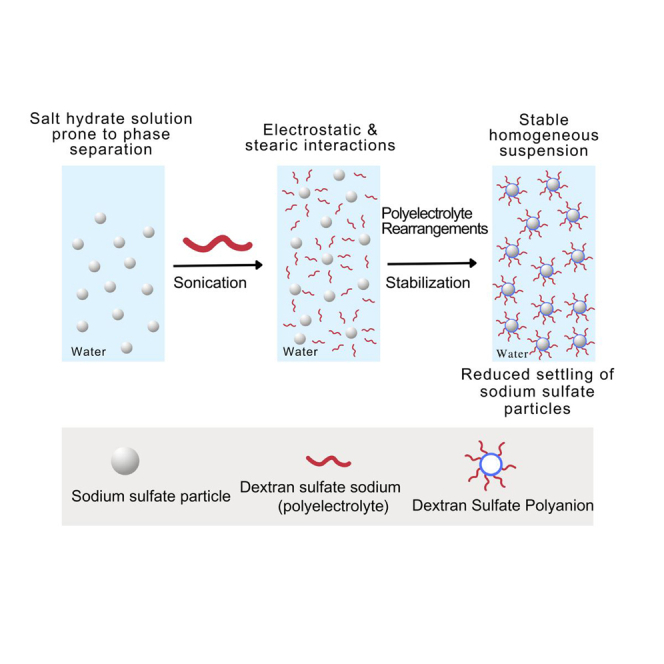

## Introduction

In a bid to reduce mismatch between energy supply and demand created by urbanization, labor productivity, industrialization, and the depletion of conventional fossil fuels, research and development of technologies with steadier, efficient, and stable energy are gaining important attention.^1^ Latent heat thermal energy storage systems (LHTESSs) are proving to be among the most efficient means of storing energy for either immediate or future usage in heating, ventilation, and air conditioning (HVAC) devices in commercial and residential buildings.^2^ Phase change materials (PCMs) have shown enormous potential for LHTESS. PCMs undergo a phase transition when subjected to an increase or decrease in temperature. They may change phase from one state of matter to the other by accumulating latent heat, while maintaining temperature equilibrium during the process.^3^ Since these materials maintain the same temperature while accumulating and discharging heat, they are promising in mitigating daily variations in solar temperature, shifting peak demands, and storing sizable amount of solar energy and other forms of renewable energy. In addition, PCMs have a large volumetric energy storage capacity (ESC), making them ideal for a variety of applications, including those in buildings, industrial waste recovery, car heat management, thermoelectric power production, and diurnal or seasonal energy storage.^4^ In buildings, the use of PCMs can smooth temperature fluctuations, reduce the peak load, and increase the thermal inertia of the building. Further, PCMs can flexibly be incorporated into either passive or active systems.^5^

Salt hydrate PCMs are highly desirable materials for heat storage applications because of their low cost, relatively low melting point, large volumetric ESC, small temperature glide, and wide availability.^6^ However, a majority of salt hydrate PCMs suffer from phase separation after successive melt-freeze cycle and supercooling that reduces the ESC. Several methods have been adopted to reduce phase separation, such as mechanical agitation of the PCM container,^7^ eutectic mixtures of two salt hydrates in appropriate concentration enabling stability,^8^ physical and/or chemical micro encapsulation of PCM, and addition of polymeric or other highly viscous materials as stabilizer. These additives have shown promise to prevent phase separation in PCM by limiting the diffusion rate and distance between the salt and water molecules during incongruent melting.^9^

In thermal energy storage (TES) applications, sodium sulfate decahydrate (SSD), Na_2_SO_4_^.^10H_2_O (Glauber’s salt), is of value because of its low cost and non-flammability. However, SSD suffers from severe phase separation and supercooling.^10^ Supercooling in SSD can be reduced through the addition of sodium tetraborate (borax) in varying concentrations.^11^ Early investigations by Marks et al.^12^ on the thermal stability of attapulgite clay-thickened SSD exhibited 320 thermal cycles. Calorimetric measurements have shown a decline in ESC of neat SSD from 238 J g^−1^ to ∼63 J g^−1^ after 40 cycles, while the thickened SSD showed a decline from 202 J g^−1^ to 105 J g^−1^ after the 200^th^ cycle. Ryu et al.^9^ observed that 2.9 wt % super-absorbent polymer (SAP) thickeners on SSD prevented phase separation for more than 300 cycles at ESC of 227 J g^−1^ on the first cycle. Gok and Paksoy^13^ investigated the effect of gelation on SSD with polyacrylamide and gelatin gels and showed that with 10 wt % gelatin and polyacrylamide 113 J g^−1^ and 180 J g^−1^ of ESC can be achieved, respectively.

Several other thickening mechanisms have also been reported in earlier studies. Li et al.^14^ investigated the effect of 2 wt % carboxyl methyl cellulose (CMC)- and 5 wt % octyl phenol polyoxyethylene ether (OP-10)-thickened SSD in 7 wt % expanded graphite (EG). They reported the initial ESC of 114 J g^−1^ with less than 9.16% degradation over 50 cycles using differential scanning calorimetry (DSC) analysis. Dong et al.^15^ reported that SSD with 9 wt % EG was able to achieve an ESC of 230 J g^−1^ in the initial cycle, which reduced to 186 J g^−1^ after 500 cycles. Alkan et al.^16^ investigated the encapsulation of SSD in hydrophilic polyvinyl alcohol (PVA) to reduce phase separation and reported an ESC of 248.7 J g^−1^ with 9 wt % PVA. Similarly, Zhang et al.^17^ showed that microencapsulated SSD in a silicon dioxide shell could achieve ESC of 125.6 J g^−1^ at the initial cycle, which then reduced to 100.9 J g^−1^ after 100 cycles. From this discussion, it is evident that most of the literature on thickening agents on salt hydrate composites was focused on preventing phase separation, either by addition of thickeners or by encapsulating the salt with polymers. So far, there has been no substantial discussion on the impact of rheological properties on the thermodynamical behavior of the thickened salt hydrate PCMs. Rheological properties, such as viscosity, storage modulus, and loss modulus of PCM composites can drastically alter the stabilization, phase separation, and supercooling in salt hydrate PCMs. Hence, there is an immediate need to fully understand the correlation between rheological properties and the fundamental mechanism of long-term stability in salt hydrate systems.^2^ Also, the reliability of thickeners and stabilizers for PCMs is worth studying as it can enable improvement in PCM design, tunability, and performance enhancement.

For long-term SSD performance, a superior stabilizing agent may be polyelectrolytes which stabilize particles in aqueous solutions via steric and/or electrostatic interactions. Low-cost polyelectrolytes are ubiquitous as additives in nanotechnology applications to stabilize nanoparticles in aqueous or organic solvents.^18^ However, little research has been done on using this approach to obtain stability in salt hydrate PCMs including SSD. Recently, Li et al.^19^ reported the stabilization of SSD using dextran sulfate sodium (DSS), a sulfated polyelectrolyte which provided stability enhancement of the thermal properties of SSD up to 150 cycles.

In the present work, eight different additives were tested as stabilizing materials to improve the long-term TES stability of SSD. The thickeners investigated in this study are sodium polyacrylate (SPA),^20^ cellulose nanofiber (CNF),^21^ potassium polyacrylate (PPA),^22^ hydroxyl ethyl cellulose (HEC),^23^ CMC,^24^ fumed silica,^25^ poly(sodium 4-styrenesulfonate) (PSS),^26^ and DSS.^19^ A thorough understanding of the thickening and stabilization mechanisms was developed by investigating the phase stability and thermophysical properties of the PCM mixtures containing SSD and one of the eight stabilizing agents. Furthermore, the structural and rheological properties of SSD with each thickener and stabilizer were investigated in detail. The correlation between rheological properties and the stabilization mechanisms was examined as a function of different thickening agents. It is observed that the rheological property changes due to the addition of the additives. Also, the additives affected the key thermophysical characteristics of SSD, including cyclic stability, phase change temperature, and latent heat. This work provides a pathway to the design and selection of additives for improving the performance of salt hydrate PCMs in TES applications, especially in building systems.

## Results

### Phase stability of the modified PCMs

**Figure 1:**
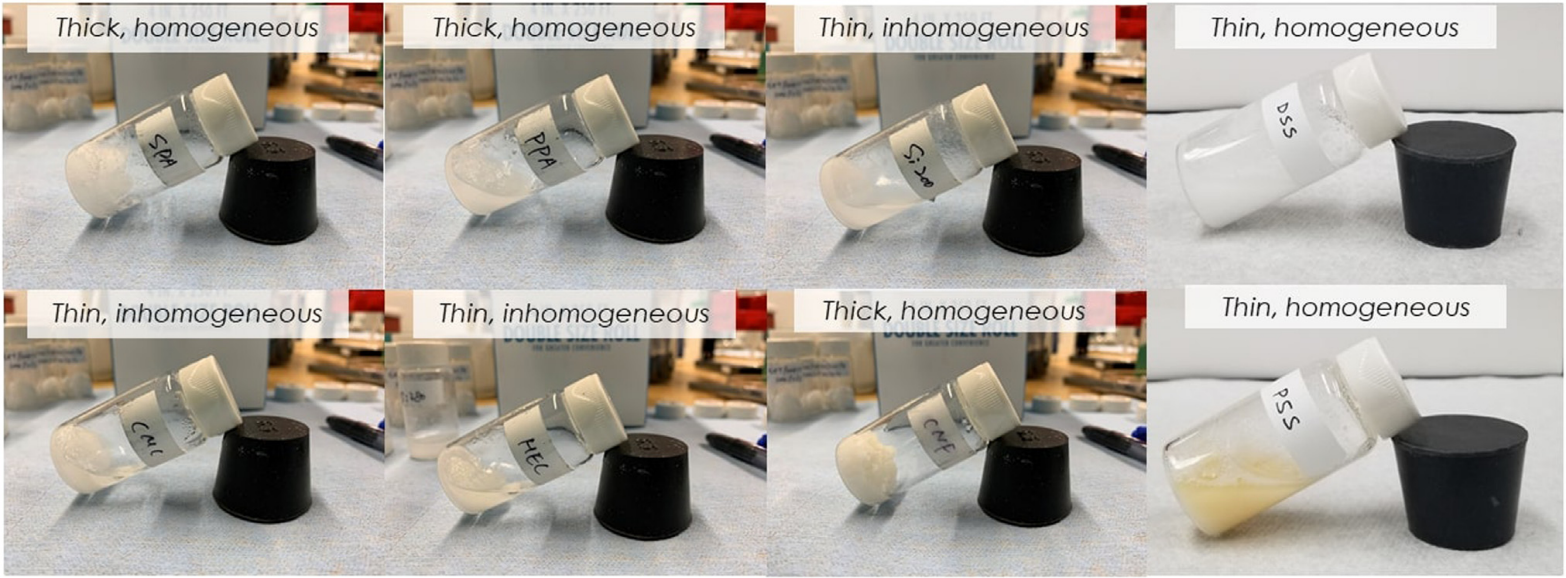
Visual inspection of viscosity and miscibility of sodium sulfate decahydrate with different thickeners and stabilizers (A) SPA, (B) PPA, (C) SiO_2_, (D) DSS, (E) CMC, (F) HEC, (G) CNF, and (H) PSS.

The effectiveness of the 8 different additives on the viscosity and miscibility of SSD was visually inspected first as presented in Figure 1. Different thickeners exhibited different thickening efficiency (thin or thick) and compatibility with sodium and sulfate ions from the molten SSD (homogeneous or inhomogeneous). Based on visual observations, only samples with SPA, PPA, CNF, and DSS and PSS showed miscibility and resulted in a homogeneous mixture. Therefore, only these 5 samples and pure SSD were further evaluated for the ESC and melting and freezing behavior in a DSC setup. Their molecular structure is shown in Figure S1 of the supplemental information.

### Structural properties of the modified PCMs

#### XRD characterization

**Figure 2:**
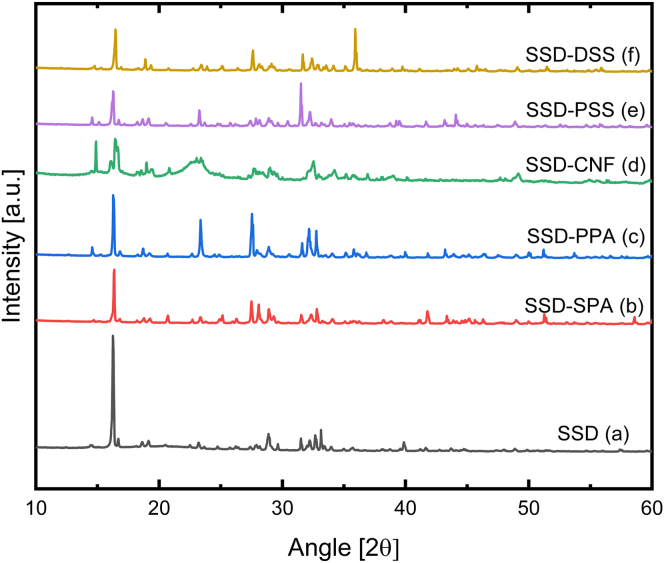
XRD Patterns of pure SSD and SSD-based PCM mixtures

The XRD diffractograms in Figure 2 represent the crystalline structures of six different PCM samples. Pure SSD showed characteristic peaks at 2θ = 16.2°, 18.6°, 23.2°, 26.2°, 27.32°, 27.9°, 31.5°, 35.7°, and 43.1°.^27^ The diffraction peaks of SSD at 16.2, 18.6, 27.32, and 27.9° correlate with the crystal planes (200), (021), (131), and (104), respectively.^17^ Furthermore, in all the diffractograms of the composite samples (Figures 2B–2F), the peak intensities were lower than the intensity of pure SSD; this is due to the interaction or coating of the crystalline SSD by the additives CNF, SPA, PPA, DSS, and PSS. The presence of crystalline peaks of pure SSD in the samples signifies that the interaction between SSD and the additives is a physical interaction, in particular electrostatic interactions, i.e., no chemical reaction occurs. The reduced intensity and broad peak in Figure 2D of SSD-CNF sample at 16.5° and 22.6° suggest the appearance of reduced crystallinity and possible preferential crystal orientation of the mixture due to the long aspect ratio of CNF.^28^ These prominences are indicative of a cellulose type I structure, and they lie on the (110) and (200) crystal planes.^29^ The (110) and (200) crystal planes also represent the presence of both hydrophilic and hydrophobic surfaces in CNF.^28^ Furthermore, the interplanar d-spacing (Å) and crystal size (nm) were calculated using Equations 1 and 2, respectively.^30^

(Equation 1) nλ=2dsinθ

(Equation 2) $$D=\frac{k\lambda }{\beta \;\cos\;\theta }$$

where D is the size of the crystal (nm), λ is the wavelength of the incoming X-ray, k is the constant dimension shape factor, k = 0.9, *β* is the full width half maximum (FWHM) in radians, and θ is the diffracted Bragg angle. The crystallography data of SSD and the PCM mixtures are reported in Table S1 of the supplemental information. The crystal size of the SSD varies between 47 nm and 284 nm.

#### ATR-IR analysis

**Figure 3:**
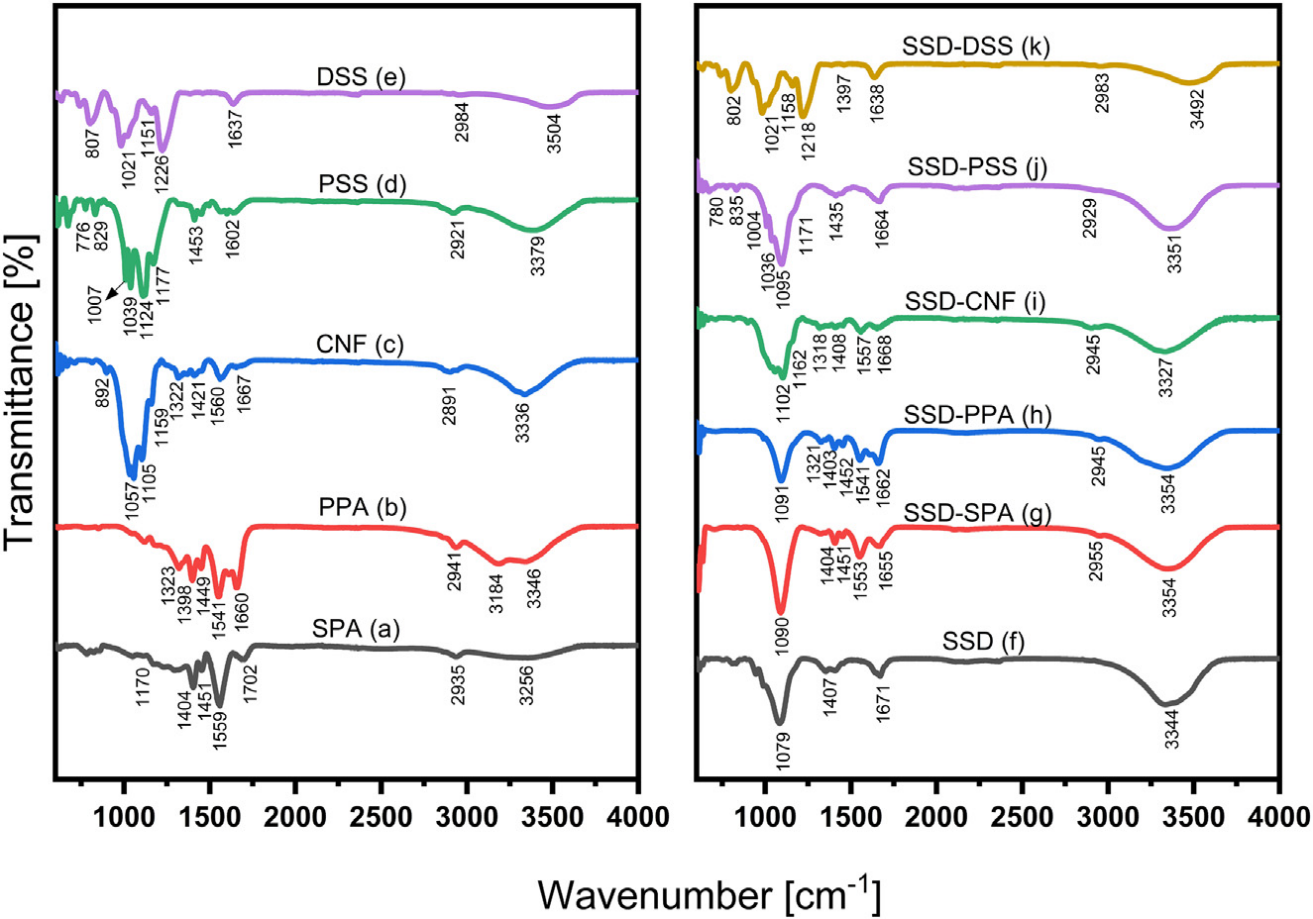
Chemical structure characterization of PCM mixtures ATR-IR Spectra of additives and PCM mixtures (A) SPA (B) PPA (C) CNF (D) PSS (E) DSS (F) SSD (G) SSD-SPA (H) SSD-PPA (I) SSD-CNF (J) SSD-PSS (K) SSD-DSS.

The attenuated total reflectance infrared spectroscopy (ATR-IR) spectra (shown in Figure 3) were utilized to characterize the basis materials and PCM mixtures. Figure 3A displays the ATR-IR spectra of SPA polymer. The peak at 1170 cm^−1^ represents the carboxylic stretching, C=O, and the -CH bending vibration is represented by 1404 and 1451 cm^−1^ peaks.^31^ At 1559 cm^−1^, we see a peak associated with C=O anti-symmetric stretching, and at 1702 cm^−1^, we see a peak associated with C=O stretching. 2935 cm^−1^ is asymmetric -CH_2_ bond stretching, whereas 3256 cm^−1^ is OH stretching.^32^ Figure 3B shows the spectra of PPA polymer. The OH stretching vibrations are represented by the 1323 cm^−1^ and 3346 cm^−1^ peaks, while the peaks at 1398 cm^−1^ and 1541 cm^−1^ are COO- radicals, representing the acrylate group.^33^ The C=O and -CH_2_ asymmetric stretching peaks occur at 1660 and 2941 cm^−1^, respectively. Also, the multi-molecular interaction of H_2_O causes the 3184 cm^−1^ absorption.^34^ The peak at 892 cm^−1^ in CNF’s ATR-IR spectrum (Figure 3C) is due to the β-glycosidic linkage in cellulose, while the peak at 1057 cm^−1^ is related to the C—O—C pyranose ring skeleton.^35^ The absorbance at 1105 cm^−1^ is attributable to the glucose ring in cellulose, while the peak at 1159 cm^−1^ shows asymmetric stretching in C—O—C linkage.^36^ CNF wagging symmetric bending and CH_2_ asymmetric bending have maxima at 1421 cm^−1^ and 1322 cm^−1^, respectively.

The aromatic ring bond extension is represented by the peak at 1560 cm^−1^, while the CH stretching vibration is represented by the peak at 2891 cm^−1^. The peaks at 1667 cm^−1^ and 3336 cm^−1^ represent water stretching.^37^ Figure 3D shows the PSS spectra; the absorption at 776 and 829 cm^−1^ is due to aromatic ring vibrations. 1007 cm^−1^ and 1039 cm^−1^ are S—O bond stretching peaks, whereas 1124 cm^−1^ and 1177 cm^−1^ are CS bond stretching peaks.^38^ 1453 cm^−1^, 2921 cm^−1^, and 1602 cm^−1^ are CH, CH bending and C=C stretching peaks, respectively.^39^ The signal at 3379 cm^−1^ corresponds to H_2_O moieties due to the hygroscopic nature of PSS.^38^ Figure 3E displays the spectra of DSS; the signals at 807 and 1021 cm^−1^ represent S—O—C and C—O—C stretching vibrations of the pyranose ring, respectively.^40^

The peaks 1151 cm^−1^ and 1226 cm^−1^ indicate the sulfate group and —SO_3_^−^ asymmetric vibration, respectively. 1637 cm^−1^ is the C—O—C deformation vibration, and 2984 cm^−1^ is C=H alkene stretching.^41^ The peak at 3504 cm^−1^ represents the hydroxyl bonding. Figure 3F shows the spectrum of SSD. The signals at 1079 and 1407 cm^−1^ indicate the asymmetric and symmetric stretching vibrations of S—O in SO_4_^34^, respectively. The signals at 1671 cm^−1^ and 3344 cm^−1^ are ascribed to the bending vibration and stretching vibration of water, respectively. The respective base materials are well represented in the spectra of the composite materials as shown in Figures 3G–3K without additional peaks. This further confirms the physical mixing of the baseline additive materials with SSD instead of new production formation due to chemical reaction.

### Rheological properties of the modified PCMs

The rheological properties, flow behavior, viscosity, storage moduli (*G′*), and loss modulus (*G″*) of modified salt hydrate PCMs were examined to understand the impact of the various additives on the viscoelastic properties of the SSD salt.

#### Flow behavior

**Figure 4:**
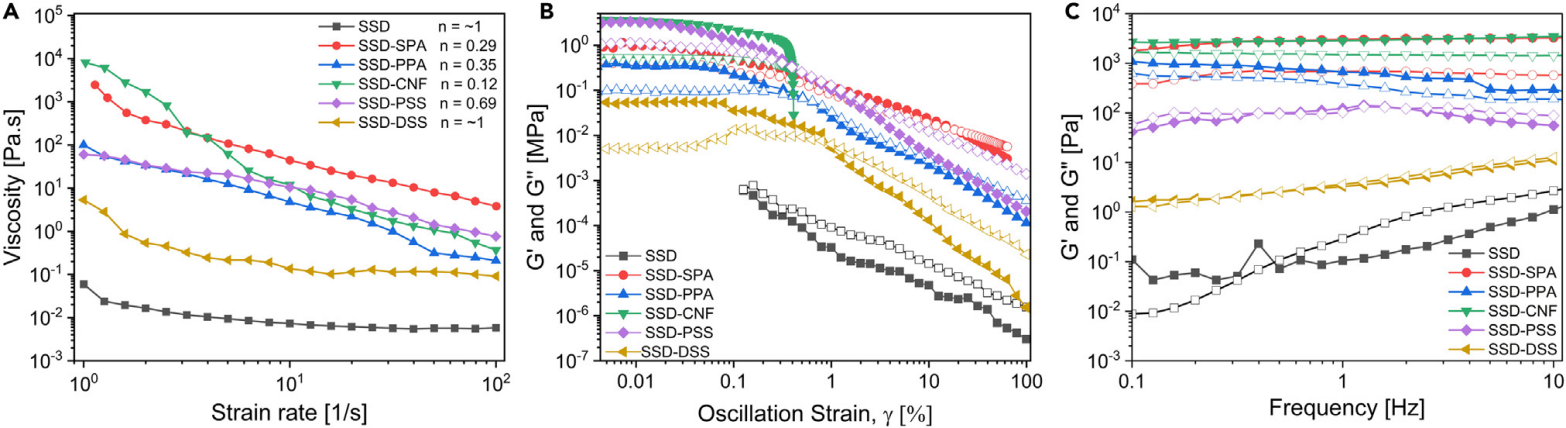
Rheology of PCM mixtures Rheological characterization of SSD-based PCM mixtures (A) Flow test (B) Amplitude sweep test. (C) Frequency sweep test. (In (B) and (C), the solid symbols represent storage modulus (G′), while outlined symbols are used for the loss modulus (G'')).

A flow sweep test was used to determine the influence of strain on the viscosity of the different mixtures as shown in Figure 4A. For the pure SSD sample, the viscosity is relatively constant with the strain rate exhibiting Newtonian behavior (Table S2). However, with the additives, shear thinning behavior was observed in the mixtures with SPA, PPA, CNF, and PSS. Viscosity follows the well-established power law as shown below^42^:

(Equation 3) $$\eta =K\gamma^{n-1}$$

where η is the viscosity, γ is the strain rate, *K* is the flow consistency index, and *n* is the power-law index. Each individual viscosity data value in Figure 4A is fitted with Equation 3, and the parameters are shown in Table 1. An “n” value less than one represents the pseudoplastic and thixotropic nature of the mixtures. As can be seen that the power-law index of n < 1 for all of the mixtures shown in Figure 4A (*n* values are listed in the figure legend), therefore, all of these PCM composites are pseudoplastic and thixotropic. As the shear rate increases, viscosity decreases, and the gel network in the polyacrylate-based composite (red circles) slowly breaks down, leading to the further decrease in the viscosity.^43^ For the CNF-based composite, the increased shearing of the material leads to the disintegration of the entangled fiber network thereby causing a reduction in the viscosity under continuous shearing.^44^ Likewise, the PSS-based mixture undergoes a breakdown in structure and decreased viscosity and flows readily in the direction of increasing. Surprisingly, the viscosity of the DSS-modified sample was relatively greater than that of pure SSD, and it exhibited Newtonian behavior, with constant viscosity value under increasing shear rate (Figure 4A). The relatively smaller increase in viscosity signifies that the stabilization mechanism of the SSD mixture with DSS is likely not achieved by the same gel formation or network breaking mechanism as seen in SPA-, PPA-, and CNF-modified PCM composites. The addition of thickeners like SPA, PPA, and CNF changed the behavior of the salt hydrate from Newtonian behavior to thixotropic behavior.^19^ Considering the degree of non-Newtonian behavior of the mixtures, as the viscosity of the materials increases, the flow consistency index, K, increases. Also, as the shear thinning increases, the power-law index decreases. Based on these, the degree of non-Newtonian characteristics of the PCM mixtures is categorized as SSD-CNF > SSD-SPA > SSD-PPA > SSD-PSS.

**Table 1:** Flow and Viscoelastic behaviors of the PCM mixtures

Sample	Flow consistency index, K (Pa.s^n^)	Power-law index, n	Critical strain, $\gamma_{c}$ [%]	G' at $\gamma_{c}$ [MPa]	G″ at $\gamma_{c}$ [MPa]	Crossover strain [%]
SSD	0.02	∼1	–	–	–	–
SSD-SPA	1120.60	0.607	0.2253	0.3552	0.2303	18.3073
SSD-PPA	96.90	0.650	0.1143	0.1993	0.0967	0.6442
SSD-CNF	1351.60	0.123	0.0467	2.7077	0.5073	0.3937
SSD-PSS	79.49	0.790	0.0714	1.5128	0.8277	0.6277
SSD-DSS	1.10	∼1	0.1965	0.0297	0.0101	1.0016

#### Viscoelastic behavior

Oscillatory experiments at 40°C were performed to understand the viscoelastic behavior of SSD with various additives. Since material stability of PCMs is an important design element for specific TES systems, the storage (*G′*) and loss modulus (*G″*) measurements could provide insight into the form stability of the PCM composites.^45^ *G′* and *G″* represent the solid- and liquid-like behaviors in amplitude and frequency sweep experiments shown in in Figure 4B and in Figure 4C. The critical strain, $\gamma_{c}$, is referred to the strain at which the material begins to yield, which is the beginning of deviation from linear viscoelasticity. Also, the strain beyond which the material changes from solid-like behavior to liquid-like behavior (*G'' > G′*) is referred to as the crossover strain. The critical strain is obtained by finding strain value of the intersection between the line drawn along the *G′* value at low strain values (where *G′* is almost constant) and the line drawn along the decreasing *G′* values at high strain values. This is demonstrated in Figure S2 of the supplemental information.

The amplitude sweeps results for SSD and composites are illustrated in in Figure 4B. Pure SSD experienced a monotonic decrease in *G″* and *G′* even at low strain levels. Furthermore, the loss modulus, *G''* (outlined symbol) is higher than the storage modulus, *G'* (closed symbol), thereby confirming the liquid or melted state of pure SSD. Because of low G′ and G″ values of pure SSD, very low strain rate values are not obtained. Interestingly, the SSD-CNF composite exhibited the opposite behavior (*G'* > *G″*) indicating a solid-like behavior. However, the composite yielded at low strain level, $\gamma_{c}$ = 4.67 x 10^−2^, before *G′* dropped rapidly. This might be attributed to the breakdown of the fibrillated cellulosic structure in the composite as observed earlier by Shel et al.^46^ Furthermore, the relevant viscoelastic parameters including the critical strain (yield point of the material) and crossover strain of the mixtures are extracted from the amplitude sweep results as shown in Table 1. SSD-SPA showed the highest critical strain and crossover strain among the mixtures. This is due to the ability of SPA polymer chains to crosslink and form a strong polymeric network with the oppositely charged salt ions that does not yield easily.^47^ This property can enable its use in forming a stable PCM matrix. With relatively lower *G′* and *G″* compared to the mixtures with thickeners, SSD-DSS mixture has the second highest critical strain and crossover strain. SSD-PPA and SSD-PSS have intermediate G′ values but low critical strain and crossover strain values compared to the mixtures with DSS and SPA.

The loss modulus exhibits higher values than the storage modulus in the frequency sweep *G′, G″* measurements as shown in Figure 4C. This represents a dominant liquid behavior. The increase in the *G′* and *G″* values of pure SSD with frequency corresponds to a well-known liquid-like relaxation behavior of the material.^45^ However, other mixtures show hardly any change in the *G′* and *G″* values with frequency, which corresponds to linear viscoelastic behavior in the samples as consistent with the amplitude sweep test results (in Figure 4B). Also, the *G′* values of the mixtures with polymeric additives (SSD-SPA, SSD-PPA, and SSD-CNF) are greater than the corresponding *G″* values over the test frequency range, signifying solid-like characteristics with elastic properties dominating over the viscous properties. In these polymeric systems, there is a formation of strong, interconnected polymeric network which restricts the movement of the polymer chains and the salt hydrate particles in the system, hence resulting in the frequency-independent response.^48^

#### Temperature ramp test

**Figure 5:**
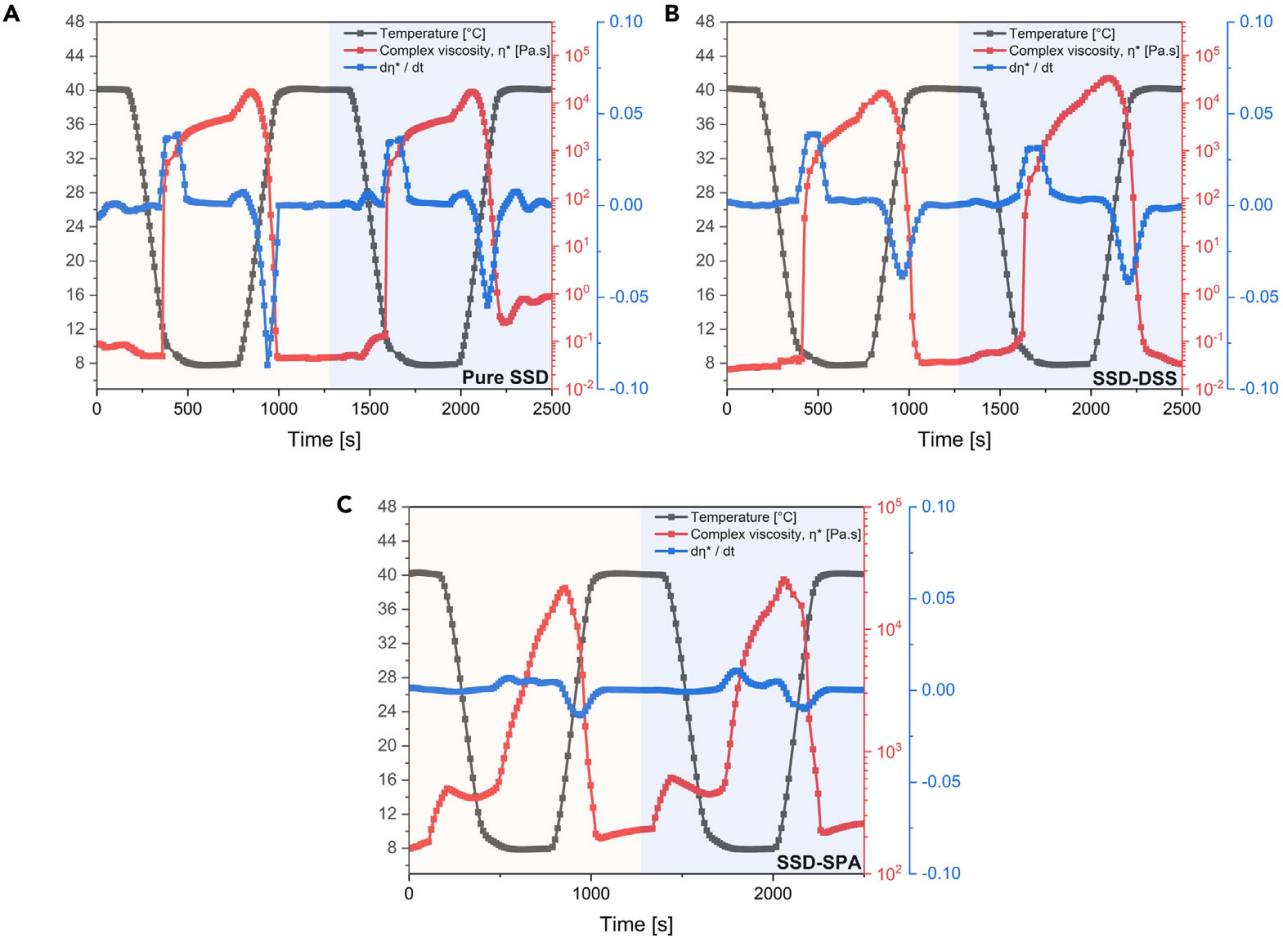
Melt-freeze transition of PCM mixtures Change in complex viscosity over the temperature range of 8°C–40°C during 2 cycles for (A) Pure SSD (B) SSD-DSS (C) SSD-SPA.

Utilizing rheology, the effect of PCM phase transition on rheological properties was evaluated to identify any potential changes that might occur through thermal cycling. To understand the impact of rheological properties on the phase transition of the composites, three samples, pure SSD, SSD-DSS, and SSD-SPA, were subjected to two thermal (heating and cooling) cycles between 8°C and 40°C as represented in Figure 5. It should be noted that the SSD-thickener mixtures have a nominal melting temperature of 32.4°C. The materials were subjected to shear strain of 1% and frequency of 1Hz; then the evolution of the complex viscosity was investigated.

Figure 5A illustrates the change in complex viscosity of pure SSD between 8 and 40°C. During the cooling step (40°C–8°C), the complex viscosity of the pure salt hydrate PCM exhibited a remarkable increase. At 10°C, and ∼390 s, the value increased by more than five orders of magnitude. This increase is also illustrated by the sharp rise in the derivative of the complex viscosity, $\frac{d\eta }{dt}$. This suggests that at this temperature, the material exhibited crystallization. However, there was an initial rise in the complex viscosity at the beginning of the heating step (8°C –40°C), as opposed to a drop. A plausible explanation is that as oscillatory strains are applied during the heating process, there may be a tendency to first break up the solid SSD particles formed during subcooling at 8°C. As higher temperatures are applied to the small SSD sample, initial melting will cause liquid to begin to flow into the subcooled particles, and refreezing could occur. This could lead to a greater overall stiffness and increase in the complex viscosity. Continuously heating the material over its melting point resulted in a considerable reduction in complex viscosity. Also, in Figure 5B, the complex viscosity increased significantly when the SSD-DSS mixture was cooled from 40°C to 8°C, which corresponds to the rapid crystallization of the material during the cooling cycle. A change in complex viscosity of five orders of magnitude occurred at 10°C, and ∼450 s, which is similar to pure SSD. In addition, the complex viscosity dropped proportionally when the material was heated above the melting point. This indicates that the addition of DSS does not affect the phase transition temperature (PCT) and does not inhibit the rapid crystallization and release of stored energy in the PCM during discharge cycles. Figure 5C depicts the changes in the rheological characteristics of the SSD-SPA composite during sequential melt-freeze cycles. Unlike pure SSD, the mixture did not exhibit a sharp transition when the material was cooled from 40°C to 8°C, as shown by the low magnitude of the derivative of the complex viscosity, $\frac{d\eta }{dt}$, when compared to pure SSD and SSD-DSS. The sudden rise in viscosity at ∼180s and ∼1350s during the constant-temperature melting process can be attributed to the transient restructuring of the SPA polymer network after the melting of the SSD is completed. In addition, the change in complex viscosity was less than three orders of magnitude after maintaining the sample at 8°C for an extended period of time. The smaller relative change in complex viscosity is due to the greater average viscosity of the SSD-SPA in its molten state; the viscosity at the end of the freezing stage is very similar for all three materials. The sluggish dynamical behavior during freezing may be due to the high absorption rate and slow release of water needed for crystallization by highly hydrophilic SPA. This could inhibit crystallization and the proper release of energy stored in the material. The similar pattern is also found in other mixtures with polymeric thickeners, such as SSD-CNF (Figure S3). From this discussion, it can be concluded that the thickening agents raise the viscosity of the salt hydrate PCMs substantially, slow the diffusion of water in the sample, and hence reduces the rate of freezing and heat release during the cooling cycles. In all cases, the two heating and cooling cycles are quite similar; this demonstrates the repeatability of the phase transition in PCMs, a requirement of stability of these materials.^49^

### Thermal properties of the modified PCMs

**Figure 6:**
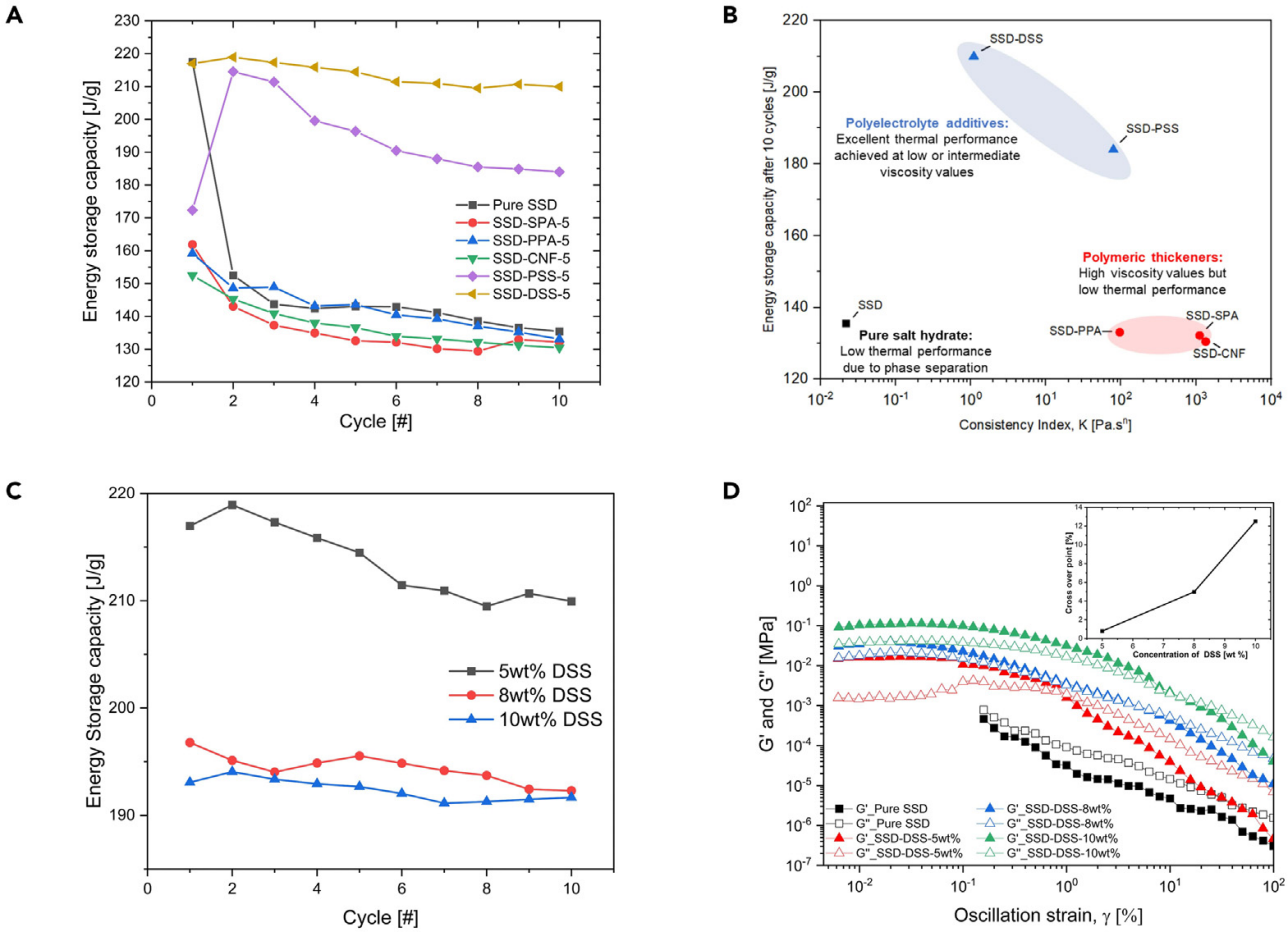
Thermal properties characterization of PCM mixtures Thermal properties of composite PCMs (A) Energy storage capacity (ESC) of pure sodium sulfate decahydrate (SSD) and SSD-based mixtures measured over 10 cycles (B) Comparison of thermal energy storage capacity vs. viscosity values of SSD and other mixtures after 10 cycles (C) ESC of SSD-DSS mixtures with various concentration of DSS over 10 cycles (D) Amplitude sweep test of pure SSD and SSD-DSS mixtures with various concentration of DSS. Inset plot shows an increase in crossover strain with corresponding increase in DSS concentration.

The influence of all five different additives on stabilizing SSD was compared. The ESC of pure SSD and the SSD mixtures was experimentally measured using DSC over 10 cycles shown in Figure 6A. The ESC of pure SSD (black curve) degraded by 38.4% (from 220 J g^−1^ to 135.21 J g^−1^) after 10 cycles. The significant decrease in latent heat of pure SSD confirmed previous findings that pure SSD is not thermally stable and that additives/thickeners are required to stabilize the salt hydrate PCM. A possible solution can be achieved by decreasing the diffusion length for the salt particles and limiting the settling rate of the anhydrous sodium sulfate in solution. Samples with sulfated polyelectrolytes, DSS (orange curve) and PSS (violet curve), showed the most desirable performance with recovered energy capacity of 209.95 J g^−1^ and 183.99 J g^−1^, respectively. However, samples thickened with SPA, PPA, and CNF experienced a decline in ESC values to 132.16 J g^−1^, 133.08 J g^−1^, and 130.47 J g^−1^, respectively, after 10 cycles.

Considering the effect of the additives on the melting point of SSD, SSD itself melts at 32.4°C, but the addition of the thickeners and polyelectrolytes raised the melting point of SSD above the melting temperature. SSD mixtures with CNF, DSS, SPA, PSS, and PPA have melting points 35.78°C, 34.99°C, 34.14°C, 34.13°C, and 32.80°C, respectively, as reported in Table 2. These additives interact with the ions in SSD and confine the water molecules from breaking away from the crystal structure easily. This leads to more energy requirement to break the bonds between the ions in SSD and water molecule, hence resulting in the increase in melting point.

**Table 2:** Phase change properties of pure SSD, with different additives

Sample	Content of SSD, β [%]	Melting Temperature [°C]	Energy storage capacity at 10th cycle [J g^−1^]	Efficient energy per unit mass of SSD, *E*_*eff*_ [J g^−1^]
SSD	100.0	32.43	135.21	135.21
SSD-SPA	95.2	34.14	132.16	138.82
SSD-PPA	95.2	32.80	133.08	139.79
SSD-CNF	95.2	35.78	130.47	137.05
SSD-PSS	95.2	34.13	183.99	193.27
SSD-DSS	95.2	34.99	209.95	220.54

The effective energy (*E*_*eff*_) per unit mass of SSD was used to determine the efficacy of SSD in various mixtures and was defined as follows^14^:

(Equation 4) $$E_{eff}=\frac{{\Delta H}_{composite}}{\beta }$$

where ${\Delta H}_{composite}$ is the melting enthalpy of composite/mixture; β represents the mass fraction of SSD in composites. It is shown in Table 2 that even after correcting the ESC in each mixture to the concentration of SSD, all samples with polymeric thickeners did not produce the desirable efficient energy per unit mass compared to the ESC of pure SSD, and also the *E*_*eff*_ values of the samples with sulfated polyelectrolytes DSS and PSS are 220.54 J g^−1^ and 193.27 J g^−1^, respectively.

The enhanced thermodynamic properties of DSS and PSS samples are due to the nature of DSS and PSS which are high-molecular-weight, water-soluble polyelectrolytes with numerous hydrophilic and negatively charged sulfonate groups which help to mitigate phase separation. Once these water-soluble polymers are dissolved in the SSD solution, they initiate an electrostatic interaction with SSD. The polyelectrolytes enclose the sodium sulfate particles and suspend them in the solution, thus reducing phase separation.^19^ However, the degradation in ESC of the samples with thickeners (SPA, PPA, and CNF) may be due to the high ionic strength of solvated thickeners. This creates imbalance in the interactions with SSD, thereby resulting in poor thermodynamic properties after repeated thermal cycles.

Secondly, thickened samples may diminish the rate of salt diffusion/solubility during the freezing process, hence lowering the ESC. Correlating the thermal performance to the flow consistency index, *K*, of the materials obtained from the flow test as shown in Figure 6B, the polyelectrolyte-based PCM mixtures showed excellent thermal performance even at low viscosity values, whereas the polymeric-thickened mixtures with high viscosity values all exhibited low thermal performance even after 10 thermal cycles. Thus, we conclude that the physical thickening alone might not enhance the total ESC of SSD after repeating thermal cycles are considered. Polyelectrolyte stabilization of SSD offers a new and promising technique for improving the thermal characteristics of SSD by electrostatic and possibly steric mechanisms at low and intermediate viscosity.

To further understand the impact of increased concentration of DSS on the thermal and rheology performance of SSD, three concentration values of DSS, 5 wt %, 8 wt %, and 10 wt %, were added to SSD. As shown in Figure 6C, the rise in the concentration of DSS resulted in a corresponding decrease in the ESC with the ESC of 5wt % DSS estimated as 213 ± 3.50 J g^−1^ as compared to 8 wt % and 10 wt % DSS with ESC of 194.39 ± 1.37 J g^−1^ and 192.38 ± 0.99 J g^−1^, respectively. The optimal concentration of DSS for higher thermal performance is 5 wt %. Figure 6D exhibits the influence of DSS concentration on the viscoelastic behavior of the mixtures. The *G′* and *G″* values of the samples increased proportionally as the DSS concentration rose from 5 wt % to 10 wt %. In addition, the inset figure demonstrates a monotonic rise in crossover strain as concentration increases.

### Effect of DSS on improving the thermal stability of SSD

**Figure 7:**
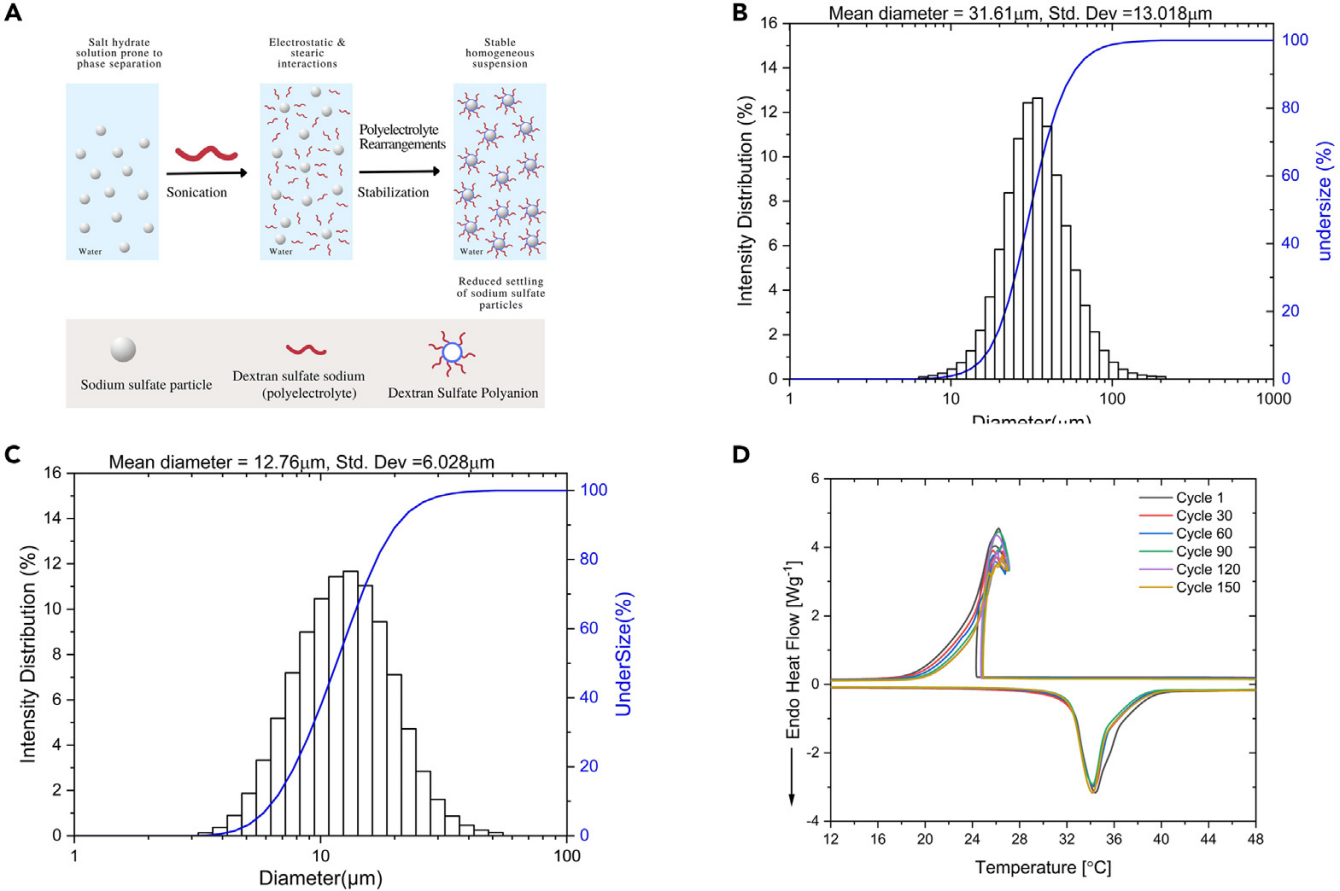
Stabilization of SSD using DSS polyelectrolyte Stabilizing effect of DSS (A) Schematic illustration of the stabilization mechanism of DSS on SSD due to electrostatic interaction and possible stearic effect. Particle size distributions of (B) Pure SSD (C) SSD-DSS mixture (D) DSC thermograms of SSD-DSS-borax through 150 thermal cycling tests. The curves are identical from cycle 1 to cycle 150 indicating the thermal stability of the PCM mixture.

In general, a desirable salt hydrate PCM must have a high thermal stability over repeated thermal cycling. The addition of DSS polyelectrolyte has provided a new proven technique to enhance the stability of SSD via polyelectrolyte stabilization. The possible stabilization mechanism is as follows: the dissolution of DSS in aqueous solution of SSD leads to sodium cations dissociating from the DSS polyelectrolytes and the formation of negatively charged dextran sulfate (DS) polyanions. A strong electrostatic contact between the sodium sulfate (SS) particles and the DS polyanion facilitates the ultimate coating of the SS particles by the DS polyanion (Figure 7A). This could lead to the formation of a homogeneous solution in which the SS particles are kept suspended in the solution like a suspension thereby reducing phase separation. This ultimately leads to increased thermal cycling stability of DSS-based PCM mixture. The stabilization mechanism is explained extensively in our previous publication.^19^

To further understand the stabilization mechanism of DSS polyelectrolyte on the particle size of SSD, dynamic light scattering (DLS) was utilized to measure the suspended particle size of SSD and SSD-DSS systems. The particle size distribution of the materials system is usually depicted in a normal distribution. As seen in Figure 7B, pure SSD has a mean particle size of 31.611 μm, but the SSD-DSS mixture has a mean particle size of 12.76 μm (Figure 7C). This indicates that the inclusion of DSS significantly reduced the mean particle size of SSD. It appears that the polyanions in DSS served as an effective shield to keep undissolved salt particles from forming big aggregates that would settle like the anhydrous salt in pure SSD. Particle/aggregate size reduction and sample homogeneity resulted in DSS-modified PCM sample having good thermal cycle stability.

### Cyclic stability test and cost analysis of DSS-modified PCM mixture

While repeated phase separation is the major limitation hindering the widespread usage of SSD as a PCM, high degree of supercooling of salt hydrate-based PCMs is also a major limitation. This results in a variation of 25°C –40°C in the freezing temperature of SSD relative to the typical melting point.^10^ Based on the promising performance of SSD-DSS mixture, PCM mixture based on SSD-DSS was prepared with sodium tetraborate decahydrate (borax) to decrease the degree of supercooling. The mixture has SSD-DSS-borax in a mass ratio of 90.8:4.6:4.6, respectively. The supercooling and *ESC* of SSD-DSS-borax mixtures during melting and freezing are summarized in Table 3. Figure 7D illustrates the thermal cycling curves for up to 150 cycles. The 1st, 30^th^, 60^th^, 90^th^, 120^th^, and 150^th^ cycles overlapped well, confirming the high thermal stability of the material. The SSD-DSS-borax exhibited a steady melting point of 34.17°C and ESC of 145.26 J g^−1^ after 150 cycles. This indicates that 77% of ESC was recovered after 150 cycles; the degradation in ESC is still minimal compared to the PCM mixtures with polymeric thickeners after repeated thermal cycles. Also, the addition of borax drastically reduced supercooling to ∼8°C. Although the freezing curves showed recalescence at the freezing peak, the level of recalescence can be reduced further by the use of fillers with high thermal conductivity, such as EG, which provide heat pathway and form a uniform heat flow in the PCM mixtures.^14^^,^^50^

**Table 3:** DSC Data for thermal cycling test of SSD-DSS-borax composite

Cycle (#)	Melting				Freezing		Supercooling (°C)
	melting temperature [°C]	Mass enthalpy [J g^−1^]	Enthalpy loss percentage (%)	Freezing temperature [°C]	Mass enthalpy [J g^−1^]	Enthalpy loss percentage (%)	
1	34.40	188.86	–	26.22	181.46	–	8.18
30	34.28	177.63	5.95	26.10	170.61	5.98	8.18
60	34.25	173.55	8.11	25.71	168.09	7.37	8.54
90	34.24	165.12	12.57	26.68	161.81	10.83	7.56
120	34.32	151.27	19.90	26.04	144.67	20.27	8.28
150	34.17	145.26	23.09	26.51	144.17	20.55	7.66

A major factor to consider in the adoption and deployment of PCMs for TES systems is cost. Based on Hirschey et al.,^51^ salt hydrate PCMs generally have low costs (0.09–2.53 $/kg), attractive melting enthalpy (100–290 J g^−1^), and competitive volumetric energy density, making them appropriate for TES purposes including building systems. Based on their evaluation, SSD is the salt hydrate with the lowest material cost (<0.09/kg); however the salt suffers from phase separation leading to instability in ESC after repeated thermal cycles. This has necessitated the addition of polyelectrolytes, such as DSS, to solve the phase separation problem. This study analyzed the cost of the DSS composite ($/KWh) to be $8.50/KWh, owing to the price of DSS (at least $10 per kilogram) which is a major material cost driver. Moreover, the cost of the stabilized PCM mixture is less than the cost target of $15/kWh set by the U.S. Department of Energy’s Building Technologies Office.^52^ Hence, the SSD-DSS-borax combination is a potential material for TES applications, particularly in building systems, because of its good thermal stability and relatively low cost.

## Discussion

In this work, the structural, thermal, and rheological behavior of SSD using different additives were investigated to improve its properties, thereby guiding future PCM materials design principles. Structural characterization using XRD and ATR-IR indicated no new phase after combining SSD with additives. Hence, it is confirmed that the mixing is a physical process without formation of new products in the PCM composite system.

The rheological analysis showed that the polyelectrolyte additives do not significantly increase the viscosity of SSD compared to the polymeric thickeners, but they induce a viscoelastic effect when added to SSD to form polyelectrolyte-based PCM composites. In addition, the temperature ramp tests demonstrate that the polyelectrolyte-based composites exhibit a fast crystallization similar to that of pure SSD. However, polymeric-thickened samples undergo slow crystallization with time, which is attributed to the sluggish kinetics in which hydrophilic thickeners limit the diffusion distance and compete with the salt for water required for the crystallization process. The poor crystallization kinetics may have a negative effect on the thermal cycling performance of PCM mixtures.

Thermal cycling results revealed that after 10 thermal cycles, polymeric-thickened mixtures with SPA, PPA, and CNF lost at least 40% of their energy storage capability. This shows that physical polymer-based thickeners alone did not prevent phase separation after repeated heat cycles. PCMs comprising DSS and PSS demonstrated thermal stability after several melt-freeze cycles with decreased phase separation. Previous findings using Zeta potential measurements show that electrostatic interaction between DSS and SSD particles coats and suspends salt particles in solution, minimizing phase separation.^19^ DLS findings revealed that DSS decreased SSD particle size, which promoted the development of a stable homogeneous suspension that prevents phase separation and increases PCM thermal cycling performance for long-term energy storage applications. We have demonstrated that the physical thickening technique alone is insufficient to improve the long-term performance of SSD; thus we propose a new path to thermal stability enhancement via electrostatic stabilization using polyelectrolyte additives with low or negligible thickening effect. While more research is needed for transitioning PCM technology from lab to market, this fundamental study showed that polyelectrolyte-based PCM composites are one of the most promising low-cost, stable materials that can be integrated to TES for cooling and heating applications in building systems.

### Limitations of the study

This study on PCMs stabilization contributes to the design and optimization of PCMs for applications in TES systems, including building structure. The thermal properties of the samples were tested using DSC, which is suitable for laboratory-scale testing. However, it utilizes minute amount of the sample (10–20mg). To investigate the performance of the materials at the system level, characterization methods such as temperature history measurements that utilize larger quantity of the samples for testing could be adopted.

## STAR★Methods

### Key resources table

**Table:** 

REAGENT or RESOURCE	SOURCE	IDENTIFIER
**Chemicals, peptides, and recombinant proteins**		

Sodium sulfate decahydrate (SSD)	Sigma Aldrich	CAS: 7727-73-3
Fumed silica (Si 200)	Evonik	CAS: 112945-52-5
Sodium polyacrylate (SPA)	Sigma Aldrich	CAS: 9003-04-7
Potassium polyacrylate (PPA)	Ward’s Chemical	CAS: 31212-13-2
Carboxymethyl cellulose (CMC)	Dow Chemicals	CAS: 9004-32-4
Cellulose nanofiber (CNF)	University of Maine	CAS: 9004-34-6
Hydroxyethyl cellulose (HEC)	Dow Chemicals	CAS: 9004-62-0
Dextran Sodium Sulfate (DSS)	VWR Chemicals	CAS: 9011-18-1
Poly(sodium 4-styrenesulfonate) (PSS)	Sigma Aldrich	CAS: 25704-18-1
Borax	Sigma Aldrich	CAS: 1303-96-4

**Software and algorithms**		

Origin 2022	OriginLab	https://www.originlab.com/
Canva 2023	Canva	https://www.canva.com/
Trios 5.2	TA Instruments	https://www.tainstruments.com/

### Resource availability

#### Lead contact

Subsequent inquiries and requests for materials and chemicals should be sent to and will be fulfilled by the lead contact, Dr. Kyle Gluesenkamp (gluesenkampk@ornl.gov)**.**

#### Material availability

No new reagents were created in this investigation.

### Method details

#### Sample synthesis

All materials were utilized as received. To overcome the difficulties of phase separation in SSD, we have developed a methodology flow chart involving materials selection, synthesis and characterization as shown in Figure S4 of the supplemental information.

The PCM mixtures were prepared by mixing SSD with the additives in a mass ratio 95.2:4.8 respectively. The samples were synthesized in a temperature- and humidity-controlled (22°C, 85% RH). Afterward, the mixtures with thickeners were stirred vigorously to achieve miscibility at high viscosity and those with polyelectrolyte additives were heated and ultrasonicated in a thermostatically controlled water bath to 50°C for 1 h, during which time the SSD melted. After letting the mixtures cool to room temperature naturally, they were stored in a tightly sealed glass vial.

#### Structural properties

##### X-Ray diffraction characterization

X-ray diffraction (XRD) was used to ascertain the structure of the SSD-based mixtures. With the use of a PANalytical Empyrean XRD diffractometer, we analyzed the XRD. There was a Cu anode with a K_α_ of 1.54 Å, an X-ray voltage of 45kV, and a tube current of 40 mA. Data for X-ray diffraction were gathered at a scan rate of 1°/min across a 2 range of 5°–90°.

##### Attenuated total reflectance infrared spectroscopy (ATR-IR)

The ATR-IR spectra of the salt hydrate samples were collected using a PerkinElmer Frontier FTIR/NIR spectrometer fitted with a diamond ATR connection and a spectral resolution of 2 cm^−1^ in the 4000-600 cm^−1^ region to identify their chemical structure.

#### Thermal properties

By employing a differential scanning calorimeter (DSC 2500, TA Instruments) under a 50 mL/min nitrogen purge, we were able to determine the phase change temperature, latent heat, and supercooling of the samples. Each sample, ranging in weight from 10 to 20 mg, was placed in a hermetically sealed aluminum DSC pan and subjected to a thermal cycling procedure at a ramp rate of 5 °C/min between −40 and 50°C. Using the heating scans, the PCM samples' melting temperatures and phase change enthalpies were calculated. The point at which freezing began during a cooling scan was compared to the melting point found during the previous melting scan to establish the existence of supercooling.

#### Rheological properties

Rotational rheometer (ARES-G2; TA Instruments) with parallel plate geometry and 40 mm diameter was used to test PCM rheological parameters. In a 1 mm space between the plates, the molten PCM samples were loaded. Water was kept from evaporating from the samples by placing them in a solvent trap. The samples were evaluated in a shear rate-controlled mode at 40°C, and the viscosity was determined as a function of shear rate from 0.1 to 100 s^−1^. In order to verify the existence of the linear viscoelastic zone, a test was performed with a stable angular frequency (1 Hz) and a range of strain from 0.005% to 100%. In oscillatory testing, the storage (G′) and loss (G″) moduli under an input fixed strain of 1% were studied for both pure SSDs and mixes including SSDs. The SSD was kept at a constant 40°C in order to maintain a liquid state during the test. In addition to the thermal cycling test, an *in situ* temperature ramp test was performed by subjecting the samples to a temperature ramping from 40°C to 8°C for two consecutive cycles.

### Quantification and statistical analysis

Origin 2022 and Trios 5.2 were used for the measurements and statistical analysis.

## Data Availability

• On request, the lead contact will share the original data reported in this article. • This article contains no original code. • Any extra data necessary to reanalyze the data given in this research is accessible upon request from the lead contact.
