# Supplementary material for: Stabilization of low-cost phase change materials for thermal energy storage applications
Source: iScience. 2023 Jun 20;26(7):107175. doi: 10.1016/j.isci.2023.107175 (PMC10329044; doi:10.1016/j.isci.2023.107175)
Supplement: Document S1. Figures S1–S4 and Tables S1 and S2 [file mmc1.pdf]

iScience, Volume *26*

Supplemental information

Stabilization of low-cost phase change materials for thermal energy storage applications

Damilola O. Akamo, Navin Kumar, Yuzhan Li, Collin Pekol, Kai Li, Monojoy Goswami, Jason Hirschey, Tim J. LaClair, David J. Keffer, Orlando Rios, and Kyle R. Gluesenkamp

SUPPLEMENTARY INFORMATION

S1 Molecular Structure of all the additives used with SSD

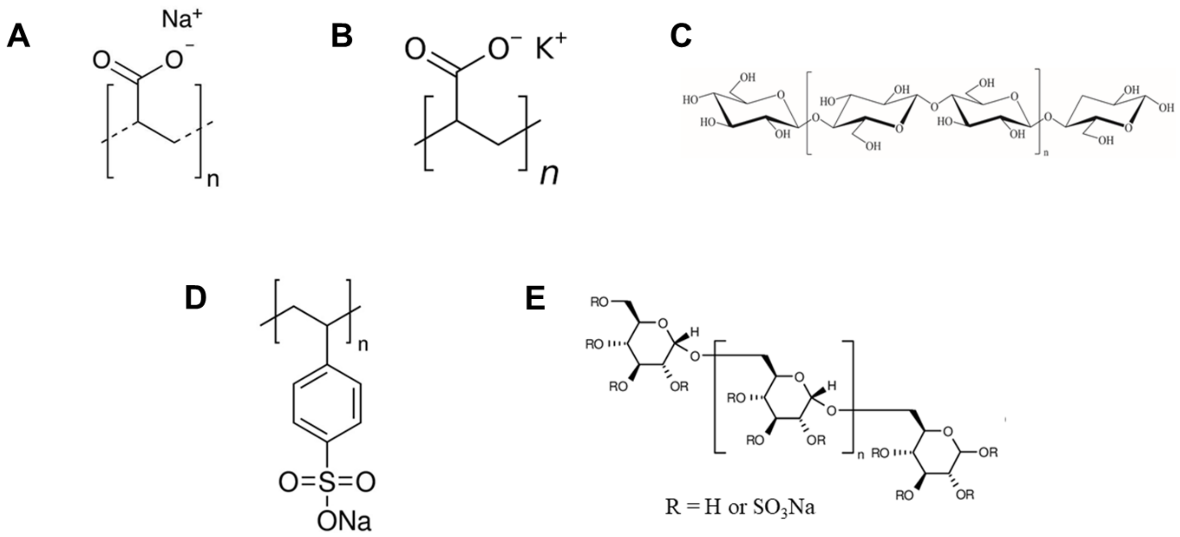

Figure S1: Molecular structures of the additives used with SSD (A) Sodium polyacrylate (SPA) (B) Potassium polyacrylate (PPA) (C) Cellulose Nanofiber (CNF) (D) Poly(sodium 4-styrenesulfonate) (PSS) (E) Dextran sulfate sodium (DSS), Related to Figure 1

S2 XRD Characterization

Table S1. Crystallography information for SSD and SSD-based PCM mixtures**,** Related to Figure 2

Samples	2θ (°)	Crystal plane (h k l)	d-spacing (Å)	FWHM (°)	Crystal size, D (nm)
SSD	16.2	(0 0 2)	5.449	0.05	167.12
	18.6	(1 2 0)	4.770	0.09	88.72
	23.2	(1 2 2)	3.830	0.08	104.20
	26.2	(1 2 2)	3.400	0.06	135.89
	27.3	(1 3 1)	3.260	0.10	80.05
	27.8	(4 0 1)	3.210	0.05	154.64
	31.5	(2 0 4)	2.847	0.03	294.41
	35.7	(3 2 2)	2.516	0.18	47.50
	43.7	(4 1 5)	2.070	0.07	127.88
SSD-SPA	16.2	(0 0 2)	5.470	0.14	58.98
	18.7	(1 2 0)	4.732	0.10	77.91
	23.3	(1 2 2)	3.809	0.10	83.85
	26.3	(1 2 2)	3.390	0.05	158.33
	27.5	(1 3 1)	3.244	0.04	223.36
	28.1	(4 0 1)	3.178	0.06	135.51
	31.5	(2 0 4)	2.836	0.04	190.56
	35.8	(3 2 2)	2.506	0.29	28.83
	43.9	(4 1 5)	2.058	0.21	41.44
SSD-PPA	16.3	(0 0 2)	5.439	0.05	152.80
	18.7	(1 2 0)	4.746	0.05	149.04
	23.4	(1 2 2)	3.805	0.05	162.83
	26.2	(1 2 2)	3.402	0.03	283.09
	27.5	(1 3 1)	3.240	0.09	89.25
	27.9	(4 0 1)	3.194	0.09	87.61
	31.5	(2 0 4)	2.831	0.03	289.57
	35.8	(3 2 2)	2.507	0.02	388.14
	43.9	(4 1 5)	2.063	0.24	36.41
SSD-CNF	16.1	(0 0 2)	5.499	0.26	30.86
	18.6	(1 2 0)	4.779	0.03	246.82
	23.4	(1 2 2)	3.797	0.47	17.06
	26.1	(1 2 2)	3.414	0.75	10.94
	27.3	(1 3 1)	3.268	0.06	143.86
	27.9	(4 0 1)	3.188	0.09	95.16
	31.7	(2 0 4)	2.821	0.09	95.99
	35.7	(3 2 2)	2.509	0.30	27.58
	43.7	(4 1 5)	2.065	0.35	24.21
SSD-PSS	16.2	(0 0 2)	5.460	0.10	77.65
	18.6	(1 2 0)	4.756	0.07	113.50
	23.7	(1 2 2)	3.754	0.05	151.10
	26.2	(1 2 2)	3.401	0.06	130.45
	27.4	(1 3 1)	3.256	0.13	64.05
	35.7	(3 2 2)	2.512	0.10	83.60
	43.7	(4 1 5)	2.071	0.10	88.65
SSD-DSS	16.4	(0 0 2)	5.394	0.08	106.98
	23.3	(1 2 2)	3.816	0.07	123.04
	26.4	(1 2 2)	3.378	0.03	278.36
	27.4	(1 3 1)	3.255	0.15	54.63
	28.1	(4 0 1)	3.175	0.15	54.828

	32.2	(2 0 4)	2.847	0.12	71.37
	35.1	(3 2 2)	2.554	0.05	163.96
	43.5	(4 1 5)	2.078	0.11	77.66

S3 Rheological behavior

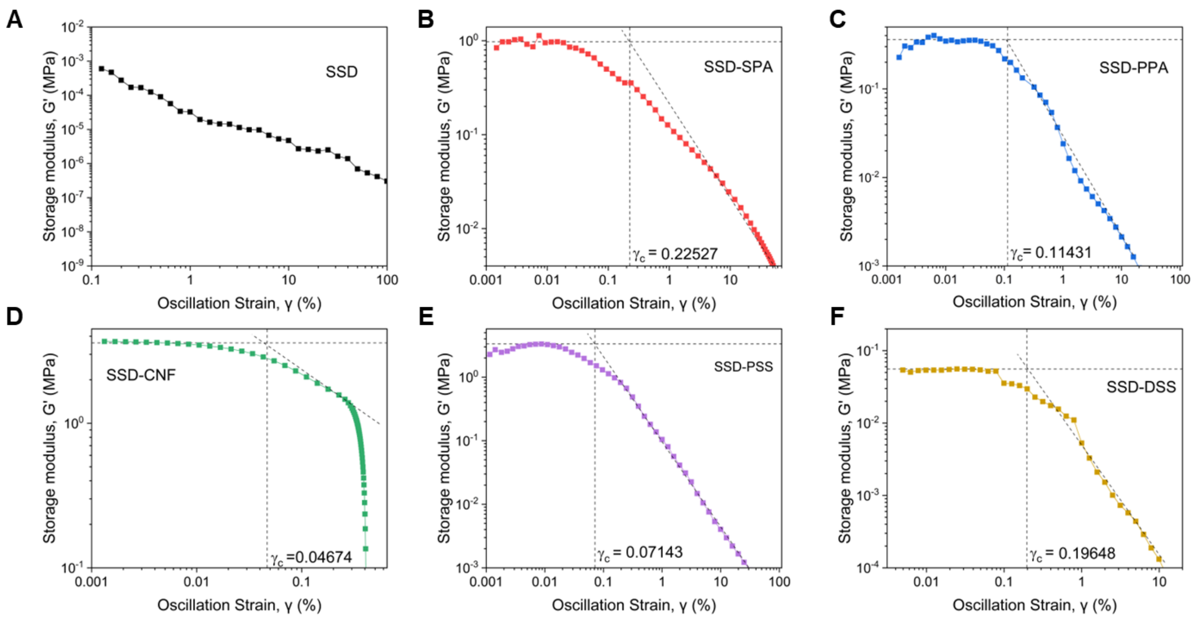

Figure S2: Critical strain determination from the amplitude sweep data for (A) pure SSD (B) SSD-SPA (C) SSD-PPA (D) SSD-CNF (E) SSD-PSS (F) SSD-DSS, Related to Figure 4

Table S2. Flow test results of pure SSD, and SSD-based composites, Related to Figure 4

Sample	Behavior	Flow Consistency Index, K ($Pa.s^{n-1}$)	Power law index, n
SSD	Newtonian	0.02	~1
SSD-SPA	Non-Newtonian	1120.60	0.607
SSD-PPA	Non-Newtonian	96.90	0.650
SSD-CNF	Non-Newtonian	1351.60	0.123
SSD-PSS	Non-Newtonian	79.49	0.790
SSD-DSS	Newtonian	1.10	~1

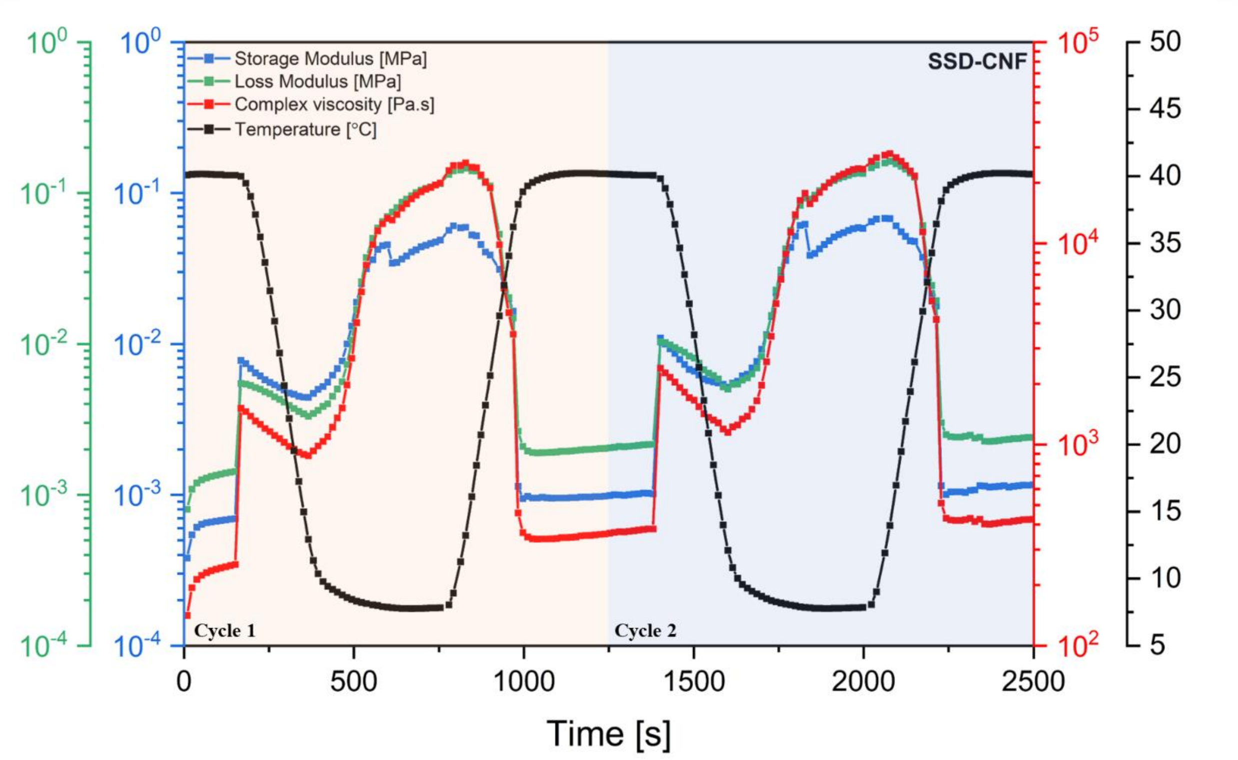

Figure S3: Change in viscosity, storage, and loss moduli over the temperature range of 8–40°C during 2 cycles for SSD-CNF, Related to Figure 5

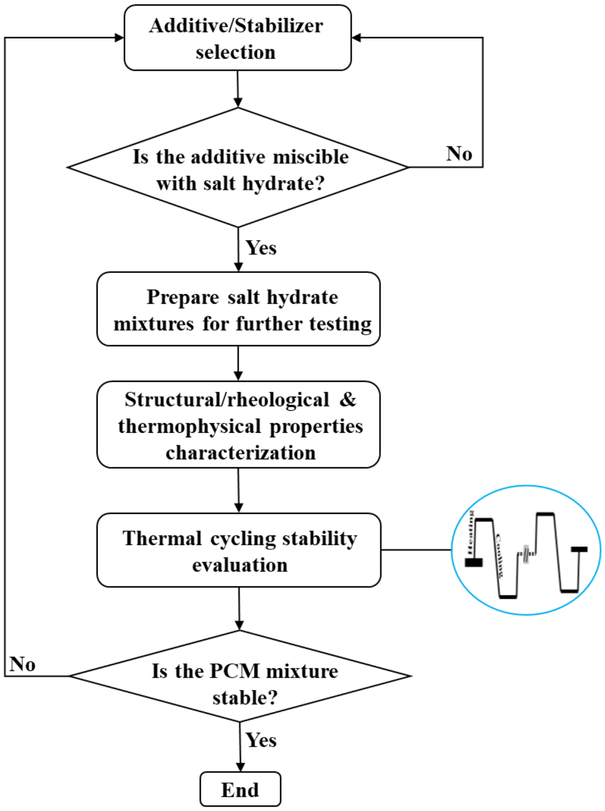

Figure S4: Methodology flow chart detailing material selection, synthesis, and Characterization. Related to the STAR Method
